# Esophageal Temperature Management in Patients Suffering from Traumatic Brain Injury

**DOI:** 10.1089/ther.2018.0034

**Published:** 2019-12-05

**Authors:** Faraaz Bhatti, Melissa Naiman, Alexander Tsarev, Erik Kulstad

**Affiliations:** ^1^ Department of Emergency Medicine, Bradford Teaching Hospitals NHS Foundation Trust, Bradford, United Kingdom.; ^2^ University of Illinois, Collaborative for Advanced Design, Research, and Evaluation, Chicago, Illinois.; ^3^ Department of Anesthesiology, Dnipropetrovsk Medical Academy of the Health Ministry of Ukraine, Dnipropetrovsk, Ukraine.; ^4^ Department of Emergency Medicine, Southwestern Medical Centre, The University of Texas, Dallas, Texas.

**Keywords:** temperature mechanisms, traumatic brain injury, brain trauma, cooling strategies, hyperthermia, targeted temperature management, esophageal temperature, heat transfer device

## Abstract

Traumatic brain injury (TBI) is a leading cause of death in the United States, and represents 2.5 million Emergency Department attendances, admissions into hospital, and deaths. A range of temperature modulating devices have been used to proactively cool TBI patients; however, there are currently no uniform targeted temperature management (TTM) guidelines in this patient population. Esophageal temperature management (ETM) is a relatively new TTM modality and the purpose of this study is to determine whether ETM is effective in controlling core temperature in TBI cases. This prospective interventional trial was a single-site study that enrolled 12 patients who received a TTM protocol using ETM. Eleven out of 12 patients reached target temperature during the first 10 hours of treatment. A total of 480 temperature measurements were recorded; 85% of the total measurements were within ±1°C of target temperature (408 measurements) and 75% were within ±0.5°C of target temperature (360 measurements). The average time to target was 5.83 ± 5.01 hours (range 1–20), with an average cooling rate of 0.58°C/h (range 0.15–1.5°C/h). This prospective interventional trial supports that ETM is a feasible TTM modality for severe TBI cases. The esophageal heat transfer device used in this study demonstrated comparable or superior performance to other commercially available TTM modalities, and the low adverse event rate may offer advantages over more invasive methods with reported higher complication rates.

## Introduction

Traumatic brain injury (TBI) is a leading cause of death in the United States, and represents 2.5 million Emergency Department attendances, admissions into hospital, and deaths (CDC, 2015; Faul and Coronado, 2015). Primary TBI leads to secondary injury over a period of time–and this can lead to blood–brain barrier dysregulation and worsening patient outcomes (Kinoshita, 2016). TBIs can be further be subdivided into minor (75%), moderate (15%), and severe (10%). Patients who survive severe brain injury are often left with life-changing neuropsychological disabilities–which approximately 90,000 people in the United States experience.

A key aim of treating this complex cohort of patients is to prevent secondary brain injury (Marion and Regasa, 2014). Pyrexia, typically defined as core body temperature of ≥38.3°C, is associated with adverse outcomes on both morbidity and mortality in TBI cases and targeted temperature management (TTM) is often used to prevent further brain insult when acetaminophen or NSAIDs fail (Polderman, 2008; Crossley *et al.*, 2014; Marion and Regasa, 2014). Potential protective mechanisms include suppressing destructive mechanisms, including excitotoxicity, neuroinflammation, spreading depolarizations, and many others (Greer *et al.*, 2008; Badjatia, 2009; Puccio *et al.*, 2009).

A range of temperature modulating devices, including cooling blankets, gel pads, and intravascular catheters, have been used to proactively cool TBI patients; however, there have been no uniform TTM guidelines in this patient population (Abou El Fadl and O'Phelan, 2017).

Esophageal temperature management (ETM) is a relatively new TTM modality, which has demonstrated effectiveness in postcardiac arrest (Markota *et al.*, 2016; Goury *et al.*, 2017; Hegazy *et al.*, 2017), non-TBI (Khan *et al.*, 2018), burn (Williams *et al.*, 2016), and refractive pyrexia cases with infectious origins (Hegazy *et al.*, 2015; Markota *et al.*, 2017). The purpose of this study is to determine whether ETM is effective in controlling core temperature in TBI cases.

## Materials and Methods

### Study design

This prospective interventional trial was a single-site study approved by the Dnipropetrovsk State Medical Academy Ethics Committee (ClinicalTrials.gov Identifier: NCT02420639). Written informed consent was obtained from each patient's next of kin or legal representative. All subjects were at least 18 years of age and diagnosed with severe TBI, for which the treating physician ordered TTM. Temperature management method was selected by physician discretion.

Patients with known esophageal deformity, evidence of esophageal trauma, known ingestion of acidic or caustic poisons within the prior 24 hours, body mass less than 40 kg, known pregnancy, terminal disease or “do not resuscitate order,” unstable hemodynamic conditions, or preexisting severe cardiac conductive disorder requiring pacing were excluded. Patients were followed up to ICU discharge or to 30 days after enrollment.

**FIG. 1.**
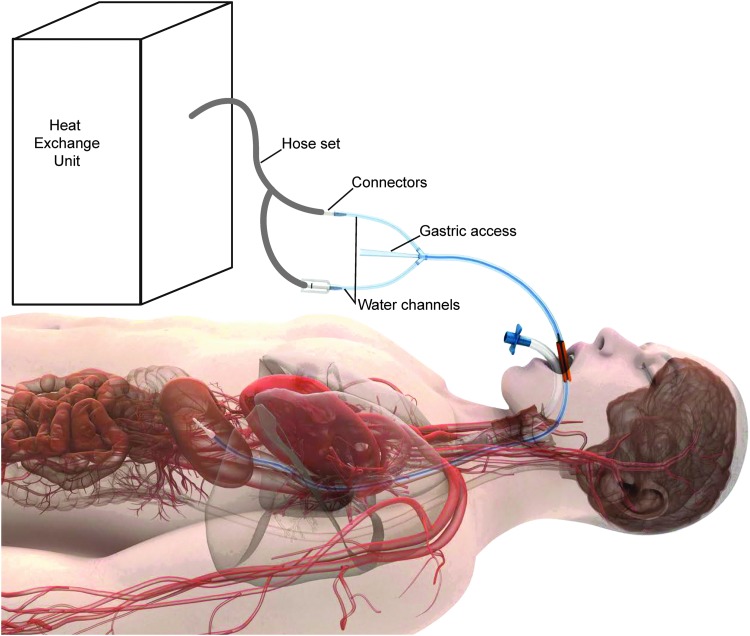
Esophageal temperature management device and system assembly.

All participants received TTM performed with an esophageal heat transfer device (EnsoETM ECD02B; Attune Medical, Chicago, IL) in accordance with its Instructions for Use (at the time of the study, the ECD02B was labeled for a 36 hour duration of use. In 2016, the ECD02 was cleared for 120 hour duration of use). In brief, the esophageal heat transfer device is a disposable, triple-lumen system that replaces a traditional orogastric tube in the esophagus (Fig. 1). Two lumens are attached to an external heat exchange unit (Blanketrol II; Cincinnati SubZero, Cincinnati, OH) and the third, independent, lumen allows gastric access. Temperature-controlled water circulates within the device to affect heat transfer; proximity to the heart and great vessels facilitates core warming or cooling.

Target temperature for the TTM protocol was assigned by provider discretion; heat exchange unit set point and water temperature were recorded hourly. Patient core temperature was measured hourly by Foley thermistor and confirmed by rectal temperature sensor readings. Adverse events specifically monitored included the following: cardiac arrhythmias, severe bradycardia, myocardial infarction/reinfarction, dysphagia, odynophagia, aspiration pneumonia, nonaspiration pneumonia, reflux, esophageal injury, and esophagitis.

## Results

A cohort of 12 severe TBI patients were enrolled between August 2015 and May 2016. Mean age was 42 ± 16 years, and 11 were male. Patient age, gender, weight, BMI, and initial GCS score are shown in Table 1. Specific diagnoses included subdural hematoma (five patients), hemorrhagic contusion (three patients), subarachnoid hemorrhage (two patients), intracranial hematoma (one patient), and epidural hematoma (one patient). Six patients underwent craniectomy.

**Table 1.** Patient Characteristics

*Patient*	*Age*	*Sex*	*Weight (kg)*	*Body mass index (BMI)*	*Glasgow coma scale (GCS)*
1	49	M	102.3	30.79	6
2	39	M	75.2	25.4	6
3	26	M	70.3	23.4	5
4	30	M	72.5	23.8	6
5	53	M	80.5	23.6	7
6	57	F	70.5	23.6	7
7	20	M	75.6	24.5	7
8	65	M	90.3	26.5	7
9	66	M	86.2	25.7	7
10	23	M	70.4	22.6	7
11	42	M	90.5	27.2	7
12	35	M	90.4	27.2	6

**FIG. 2.**
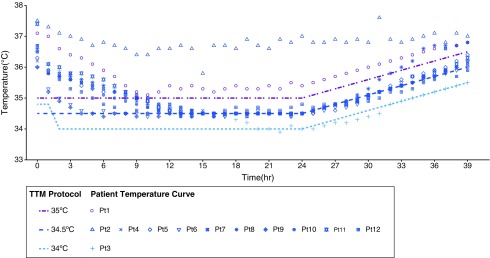
Patient temperature curves for 12 patients during treatment.

Most patients (10) were assigned a target temperature of 34.5°C, one patient was assigned a target of 35°C, and one patient was initially assigned a target of 34.8°C, which was adjusted to 34°C during the third hour and maintained for the remaining 36 hours of treatment. Figure 2 shows the TTM protocol target temperature and the temperature profile for each individual patient. Most patients received propofol and fentanyl for sedation, and six patients also received the nondepolarizing neuromuscular blocking agent pipecuronium.

Eleven out of 12 patients reached target temperature during the first 10 hours of treatment. A total of 480 temperature measurements were recorded; 85% of the total measurements were within ±1°C of target temperature (408 measurements), and 75% were within ±0.5°C of target temperature (360 measurements). The average time to target was 5.83 ± 5.01 hours (range 1–20), with an average cooling rate of 0.58°C/h (range 0.15–1.5°C/h).

Survival outcomes were not among the analysis endpoints, condition at discharge was recorded. All patients survived to discharge, with CPC scores of 1 (three patients), 2 (five patients), 3 (three patients), and 4 (one patient). One adverse event, a small volume left hydrothorax, was reported, but did not require treatment and was ultimately determined to be unrelated to the study device.

## Discussion

Although this study was not designed to make a direct comparison between ETM and other TTM modalities, its effectiveness is comparable or superior to other modalities used during TBI treatment reported in the literature. In one of the earliest studies of hypothermia for TBI, Marion *et al.* (1997) used cooling blankets and nasogastric lavage to attain goal temperature in a mean of 10 hours after injury. Clifton *et al.* (2001) used ice packs, gastric lavage with iced fluids, and temperature-control pads to achieve target temperature in 8.4 ± 3.0 hours. A study of induced normothermia in TBI patients using a triple-lumen intravascular cooling catheter attained target temperature in approximately 6 hours (interpolating from temperature graph) (Puccio *et al.*, 2009).

The National Acute Brain Injury Study: Hypothermia II compared normothermia (37°C) with two hypothermia protocols (33°C and 35°C). Patients randomized to the hypothermia group were initially cooled to 35°C with up to 2 L of cold crystalloid and application of wet sheets or gel packs and then further cooled to 33°C using chilled intravenous crystalloids, gastric lavage with cold water, and an advanced hydrogel pad system. The mean time to target was 2.6 ± 1.2 hours for patients assigned to the 35°C protocol and 4.4 ± 1.5 hours for patients assigned to the 33°C protocol (Clifton *et al.*, 2011). In the Prophylactic Hypothermia Trial to Lessen Traumatic Brain Injury–Randomized Clinical Trial (POLAR-RCT), hypothermia was induced by patient exposure, a bolus of up to 2 L intravenous ice-cold (4°C) 0.9% saline, and surface-cooling wraps to reach an initial temperature of 35°C.

Patients who were not at risk for severe bleeding were further cooled to 33°C ± 0.5°C with surface wraps. Among the 233 (89.6%) patients in the hypothermia group who reached target temperatures, median time from injury to the initial 35°C target was 2.5 hours (IQR, 0.8–5.5). Among the 186 patients (71.5%) who were assigned to the 33°C target, median time from injury to target was 10.1 hours (IQR, 6.8–15.9). Study-wide, the average time from injury to randomization was 1.9 hours (IQR, 1.0–2.7).

However, a substantial subset of patients assigned to the hypothermia group did not receive the intended treatment; 85 evaluable patients (33%) in the hypothermia group received less than 48 hours of hypothermia (33°C–35°C), and 27% of patients in the hypothermia group never reached the final target temperature of 33°C (Cooper *et al.*, 2018).

Another aspect of device performance to consider when evaluating ETM is adverse event rates. The study protocol did include active adverse event monitoring. Only one patient experienced a reportable event, a hydrothorax that did not require treatment and which was not felt to be device related.

In comparison, more invasive methods report higher complication rates, including occult or asymptomatic thrombosis, some of which are considered higher risk due to their more proximal (including caval) location (Simosa *et al.*, 2007; Prunet *et al.*, 2009; Lau *et al.*, 2010; Gierman *et al.*, 2013; Maze *et al.*, 2014; Gillon *et al.*, 2015; Reccius *et al.*, 2015; Wang *et al.*, 2018).

The thrombosis risk from endovascular cooling catheters has been suggested to be higher than standard central line intravascular catheters due to differences in materials (Wang *et al.*, 2018), or shape, where the nonuniform diameter may cause distal eddy flow, blood pooling, and stagnation, in turn, promoting activation of coagulation and the formation of clot in a more proximal location than can be easily visualized with standard compression ultrasonography (Gierman *et al.*, 2013; Gillon *et al.*, 2015; Reccius *et al.*, 2015).

One major limitation of this study is the small sample size. As is often the case in small interventional trials, the summary statistics reported in this study were heavily influenced by a single patient (Patient 2). Removing this patient from analysis could not be statistically justified, but the clinical details are noteworthy.

In addition to severe TBI, Patient 2 had significantly delayed care; over 20 minutes elapsed between the witnessed injury and ambulance arrival and almost 4 hours elapsed before advanced life support and admission to the study site while comatose. This patient's first recorded temperature, 37.5°C, was already warmer than typical in this cohort and he remained approximately 2°C above target for the first 19 hours of treatment, at which point the treating clinician elected to adjust the target temperature from 34.5°C to 36.6°C.

Cooling rates are known to be slower in patients with central fever, and studies using various cooling methods in this population report mean times to target temperature that range from 2.2 hours to over 16 hours (Diringer, 2004; Aujla *et al.*, 2017).

## Conclusions

This prospective interventional trial supports that ETM is a feasible TTM modality for severe TBI cases. As is expected in this patient population, there were challenges controlling temperature in individual cases, but overall, esophageal heat transfer demonstrated tight maintenance of goal temperature. The absence of established TTM guidelines makes device evaluation more difficult, as there is no clear benchmark for adequate performance. However, the esophageal heat transfer device used in this study demonstrated comparable or superior performance to other commercially available TTM modalities and the low adverse event rate may offer advantages over more invasive methods with reported higher complication rates.
